# Emotion Recognition and Impulsive Choice in Relation to Methamphetamine Use and Psychosis Symptoms

**DOI:** 10.3389/fpsyt.2019.00889

**Published:** 2019-12-13

**Authors:** Shalini Arunogiri, Antonio Verdejo-Garcia, Rebecca McKetin, Adam J. Rubenis, Rebecca E. Fitzpatrick, Dan I. Lubman

**Affiliations:** ^1^Turning Point, Eastern Health, Richmond, VIC, Australia; ^2^Monash Addiction Research Centre and Eastern Health Clinical School, Monash University, Box Hill, VIC, Australia; ^3^Turner Institute for Brain and Mental Health, Monash University, Clayton, VIC, Australia; ^4^National Drug and Alcohol Research Centre, University of New South Wales, Sydney, NSW, Australia

**Keywords:** methamphetamine, psychosis, social cognition, cognition, comorbidity

## Abstract

**Introduction:** The cognitive profiles of people with methamphetamine use disorder are characterized by impulsivity and impairment in social cognition. However, previous studies have not fully accounted for the presence and impact of co-occurring mental health problems on these domains. For instance, psychotic symptoms are commonly experienced by people who use methamphetamine and may influence cognitive performance. We aimed to examine decision making and emotion recognition in individuals with methamphetamine use, compared to healthy controls, to map the nature and degree of impairments in relation to the presence of psychotic symptoms.

**Method:** In this naturalistic study, we assessed reward-based decision-making and facial emotion recognition across three groups, methamphetamine-using individuals with (MAP, n = 29) and without psychotic symptoms (MNP, n = 70), and healthy controls (HC, n = 32).

**Results:** In comparison to healthy controls, methamphetamine-using individuals presented with poorer performance on tasks of decision-making and emotion recognition. Emotion recognition was impaired across all methamphetamine-using individuals, with significantly poorer recognition of anger and sadness in those with psychotic symptoms.

**Conclusion:** We found specific impairments in emotion recognition in relation to psychotic symptoms in people who use methamphetamine regularly. This builds on previous evidence on cognitive profiles in methamphetamine use disorder, highlighting the need to assess co-morbid mental health and psychotic symptoms. Our finding that methamphetamine-using individuals with psychotic symptoms present with particular difficulties recognizing anger has implications for frontline clinicians.

## Introduction

A significant public health consequence of growing global methamphetamine use is the burden of associated mental health problems (1, 2), particularly methamphetamine-related psychosis (MAP), on acute health and psychiatric inpatient services (3, 4). Observed in between 20 and 60% of individuals who use the drug regularly (5), MAP is characterized by a transient paranoia, with or without hallucinations, which is very similar in appearance to acute paranoid schizophrenia (6). MA use has been associated with psychotic symptoms in both experimental studies and during acute intoxication with illicit use (5–7), with persistent forms resembling chronic primary psychotic disorder (5, 8, 9).

Although there is a growing body of evidence characterizing the MAP syndrome, there is currently little evidence about its cognitive underpinnings (10). Cognitive markers are a promising avenue of investigation across psychotic syndromes, and present an objective, reliable means of characterizing clinical phenotype. In terms of MA use, cognition has been studied in relation to persistent versus transient MAP (8, 11). A study examining persistent MAP identified impairments in verbal learning and memory, and executive function and decision making that were comparable to those found in a chronic schizophrenia comparison group, with poorer performance compared to both healthy controls or people who used methamphetamine regularly and did not have psychosis (8). Two studies assessing cognition in transient MAP also found impairments in executive function and memory compared to healthy controls, with MAP participants and individuals with schizophrenia presenting with (similar) deficit profiles (12, 13). Notably, impairments in similar cognitive domains, particularly verbal memory, have also been demonstrated in studies investigating first episode psychosis (14), contributing to a growing body of evidence pointing to commonalities in the process of psychosis in MAP and primary psychotic disorders (5). Impairments in social cognition have also been identified across a range of primary psychotic disorders (15). Deficits in facial emotion recognition (FER), a specific domain of social cognition, have been consistently found in both ultra-high risk (for psychosis) and first episode psychosis populations (16), suggesting these impairments may be pre-existing, and independent of the stage of psychotic illness.

On the other hand, studies of cognition in MA use disorder highlight the possibility that cognitive deficits arise from prolonged drug use and related neuro-adaptation (17). A recent meta-analysis of studies of cognition in MA identified impairments in social cognition and impulsive and reward-related processes, including emotional decision-making (e.g., a preference for immediate small rewards over larger delayed rewards) (17). However, none of the studies to date have examined the impact of psychosis co-morbidity, even though sub-threshold psychotic symptoms are extremely common in people who use MA regularly. Consequently, it remains unclear whether cognitive deficits in people who use MA relate only to drug use itself, or to co-occurring psychotic symptoms.

We investigated the relationship between psychotic symptoms and cognitive impairments (including FER) in methamphetamine-using adults and healthy controls. We hypothesized that methamphetamine-using participants with past-month clinically significant psychotic symptoms (MAP) would present with cognitive impairments relative to both methamphetamine using participants without psychotic symptoms (MNP) and health controls (HC).

## Method

### Study Design, Participants and Setting

This cross-sectional study compared cognition across three groups (MAP, MNP and HC). Methamphetamine-using participants were recruited from both public and private residential alcohol and other drug treatment facilities and the community in metropolitan Melbourne, Australia between March 2015 and February 2017 (n = 99).

Inclusion criteria were (i) being aged 18 or over, (ii) at least weekly methamphetamine use in the past month, (iii) not being currently dependent on drugs (other than methamphetamine, nicotine, alcohol or cannabis), (iv) no previous diagnoses of primary psychotic disorders including schizophrenia or bipolar disorder (screened using the Structured Clinical Interview for DSM-IV TR (SCID) (18), and (v) no lifetime history of loss of consciousness for more than 30 minutes, HIV, epilepsy or any central neurological illness. Participants with previous non-psychotic psychiatric disorders were included. Age and gender matched healthy control participants (HC, n = 32), mainly students, were recruited from the same area. Participants completed informed consent and were reimbursed AU$30. Ethics approval was obtained from the Monash University Human Research Ethics Committee (CF15/40-2015000222).

## Measures

### Psychotic Symptoms

Past-month clinically-significant psychotic symptoms were defined as a score of 4 or greater on any of the Brief Psychiatric Rating Scale (BPRS) (19) positive psychotic symptom items of suspiciousness, hallucinations or unusual thought content. This method has been previously used to examine the prevalence and correlates of psychotic symptoms in studies of MA and MAP, with high inter-rater reliability reported in original studies (IRR = 0.67–0.88) (20–22). Methamphetamine-using participants were divided into those with clinically-significant past month psychotic symptoms (MAP, n = 29) and without psychotic symptoms (MNP, n = 70).

### Methamphetamine Use

Days of methamphetamine use in the past month was assessed using the Timeline Followback (23), as previous research has found has found a strong dose-response effect between days of use and psychotic symptoms (20). The TLFB is a validated measure of psychoactive substance use and shows 88% sensitivity, 96% specificity, and a 95% hit-rate and 0.77 test–retest agreement, for the use of amphetamines in the past 30 days (24). Severity of dependence on methamphetamine was assessed with the Severity of Dependence Scale (SDS), with scores ranging from 0 (low) to 15 (high) (25), with high validity and reliability in substance-dependent populations (26). Age of first methamphetamine use was based on self-report.

### Cognitive Battery

The neuropsychological test battery targeted cognitive domains associated with psychostimulant use (17) and deficits in emotion recognition associated with methamphetamine use and primary psychotic disorders (27, 28). The tasks were administered in a set order and nested within the structured interview.

### Impulsivity and Reward-Based Decision-Making

Iowa Gambling Task (IGT): a computerized task evaluating reward and punishment-based decision-making (29). The task instructs participants to try and win as much money as possible by making 100 selections of cards from four decks (A, B, C, D). Two of the decks (A and B) result in high immediate gains but in the long term will take more money than they give and can be considered ‘disadvantageous’. In contrast, two decks (C and D) have low immediate gains but will yield more money than is taken and can be considered ‘advantageous’. The outcome variable was the net score, calculated by subtracting the number of disadvantageous choices (decks A + B) from the number of advantageous choices (decks C + D) for each block of 20 trials.

### Impulsive Choice in Decision-Making

Delay Discounting Task (DDT): a measure of impulsivity in decision-making, specifically the inability to delay gratification. The task involves examining the outcome of 27 choices between smaller immediate rewards versus larger delayed rewards, based on the Kirby Monetary Choice Questionnaire (30), with the main outcome variable calculated as the k score based on methods detailed by Kirby and colleagues (30), with higher k scores indicating higher levels of impulsivity.

### Facial Emotion Recognition

The Ekman Faces Test (EFT) was used to assess FER (31). The EFT is a computerized test that presents 60 faces portraying six basic emotions (fear, anger, sadness, disgust, happiness and surprise). Dependent variables were the number of correct identifications for each emotion (ranging from 0 to 10) and total number of correct identifications (ranging from 0 to 60).

### Statistical Analysis

In order to investigate the primary hypothesis, we compared cognitive performance across all groups, using a non-parametric omnibus test (Kruskall Wallis test), and subsequent between-group differences with a post-hoc Dunn test. Confounding sociodemographic variables that were significantly different between the three groups (MAP, MNP, HC groups) were investigated using chi-squares, one-way ANOVAs for parametric variables, and Kruskal Wallis test for non-parametric variables. Drug use variables were compared between the two MA-using groups (MAP and MNP) using chi-squares and t-tests for parametric variables, and Mann–Whitney U tests for non-parametric variables.

We also examined differences in accuracy of identification of discrete emotions within the FER task (E.G., Anger, fear) based on the number of correct identifications per emotion. We compared accuracy of discrete emotion recognition between groups using a generalized linear model (GLM) to estimate the association between an individual’s group membership (MAP, MNP, HC) and correct identification of discrete emotions. In this analysis, the outcome variable was the number of trials (Out of 10) where the participant correctly identified the emotion. The outcome variable and group (MAP, MNP, HC) was the predictor variable, with HC nominated as the reference group. The model was based on a binomial distribution and a logit link function. A sandwich (robust) estimator was used to calculate the standard errors in the model, to correct for any potential lack of independence between the 10 attempts for an individual.

All tests were two-tailed with statistical significance set at p < 0.05. Statistical analyses were performed using Stata 15 (Statacorp LP, College Station, TX, USA).

## Results

The MAP (N = 29) and MNP (N = 70) groups did not differ from the healthy control group (N = 32) on any socio-demographic measures, including years of education (see **Table 1**). The two methamphetamine-using groups (MNP N = 70, MAP N = 29) did not differ on any indices of methamphetamine use (see **Table 1**). Methamphetamine use frequency was high across both the MNP group (Mean 21.7 days of use in past 28), and the MAP group (Mean 23.5 days of use in past 28), with both groups having a high severity of dependence score (MNP mean SDS 10.1, MAP mean SDS 11.2).

**Table 1:** Participant characteristics.

	HC* (n = 32)	Methamphetamine-using participants		Test statistic^1^	p-value
		MNP* (n = 70)	MAP* (n = 29)		
Male, n (%)	22 (69)	49 (69)	25 (86)	χ^2^ = 3.41	0.182
Age (mean, SD)	32.4 (1.72)	32.6 (1.03)	31.8 (1.42)	χ^2^ = 0.174	0.917
Unemployed, n (%)	17 (53)	52 (74)	22 (79)	χ^2^ = 7.17	0.306
Years of education (mean, SD)	13.0 (0.35)	13.2 (0.32)	12.3 (0.40)	χ^2^ = 2.209	0.331
IQ (mean, SD)	101.0 (1.81)	96.5 (1.35)	97.2 (2.13)	F = 1.83	0.165
**Methamphetamine and other drug use**					
Frequency of use (mean, SD)	–	21.7 (1.21)	23.5 (1.58)	z = −0.56	0.579
Age of Onset (mean, SD)	–	24.3 (1.07)	24.3 (1.82)	z = −0.036	0.972
Severity of Dependence (SDS) (mean, SD)	–	10.1 (0.43)	11.2 (0.67)	z = −1.67	0.096
Cannabis Dependence, n (%)	–	15 (21.13)	7 (24.14)	χ^2^ = 0.11	0.742
Alcohol Dependence, n (%)	–	5 (7.04)	2 (6.90)	χ^2^ = 0.00	0.979

*HC, Healthy Controls MNP; Methamphetamine use, no psychotic symptoms MAP; Methamphetamine use, psychotic symptoms.

^1^Omnibus test for comparison between three groups, chi^2^ for categorical variables and ANOVA for continuous, normally distributed; Kruskal Wallis test for continuous, non-parametric; t-test, chi^2^, or Mann–Whitney U for comparison between two groups.

In terms of cognitive performance across all three groups, there was no significant difference between verbal memory and recall (delayed recall score) between the HC (Mean 8.72 ± 1.78), MNP (Mean 8.59 ± 2.50) and MAP (8.48 ± 2.31) groups (p = 0.946). There were significant differences between groups for performance on emotion recognition, the Iowa Gambling Task and the Delay Discounting Task (**Table 2**). Post-hoc tests comparing each group revealed the HC group had significantly better performance on the Iowa Gambling Task compared to both MA using groups, with no difference between the MAP and MNP groups. For the Delay Discounting Task, post-hoc testing found significantly higher levels of impulsivity (k score) in the MNP group compared to the HC group, with no significant difference between the HC and MAP groups. Finally, for emotion recognition, the MAP group were significantly poorer at accurately identifying emotions in comparison to the MNP group and the HC group; there was no significant difference in emotion recognition performance between the MNP and HC groups.

**Table 2:** Cognitive performance across groups.

Cognitive test	Groups	Mean ± SD	P^1^	Post-hoc Test^2^					
				HC-MAP Z	P	HC- MNP Z	P	MAP- MNP Z	P
Decision-Making (Iowa Gambling Task net score)	HC MNP	22.75 ± 32.84 2.94 ± 22.34	0.006	−2.79	0.003	2.86	0.002	0.477	0.317
	MAP	0 ± 28.95							
Impulsivity (DDT k score)	HC	0.10 ± 0.11	0.027	−1.52	0.064	−2.81	0.003	0.94	0.173
	MNP	0.15 ± 0.10							
	MAP	0.14 ± 0.10							
Facial emotion recognition (Ekman’s Test Total Score)	HC MNP	46.50 ± 5.86 45.86 ± 7.53	0.007	−2.60	0.005	−0.02	0.494	3.02	0.001
	MAP	42.76 ± 4.86							

^1^Kruskal Wallis rank sum test.

^2^Post-hoc Dunn test.

In terms of accuracy of identification of discrete emotions, individuals in the MAP group were specifically impaired in recognition of anger (OR 0.56) and sadness (OR 0.57) compared to HC participants (**Table 3**). The MNP group had no significant differences in recognition of any discrete emotions with reference to the HC group.

**Table 3:** Discrete emotion recognition across groups.

	HC (n = 32)		MAP (n = 29)		MAP (n = 29)			OR (95% CI) (compared to HC group)	P value
	M	SD	M	SD	M	SD			
Anger	7.75	0.32	7.14	0.21	6.59	0.38	MNP	0.73 (9.48–1.09)	0.121
							MAP	0.56 (0.35–0.91)	0.018
Disgust	7.13	0.38	7.14	0.26	6.48	0.30	MNP	1.01 (0.65–1.56)	0.188
							MAP	0.74 (0.48–1.16)	0.972
Fear	6.44	0.41	6.45	0.27	6.00	0.47	MNP	1.01 (0.67–1.52)	0.978
							MAP	0.83 (0.50–1.38)	0.474
Happiness	9.75	0.11	9.54	0.12	9.52	0.15	MNP	0.53 (0.19–1.46)	0.216
							MAP	0.51 (0.17–1.47)	0.210
Sadness	7.13	0.36	7.04	0.28	5.86	0.33	MNP	0.96 (0.62–1.48)	0.856
							MAP	0.57 (0.37–0.88)	0.012
Surprise	8.31	0.28	8.52	0.19	8.31	0.24	MNP	1.17 (0.72–1.90)	0.528
							MAP	1.00 (0.60–1.66)	0.995

## Discussion

In this study examining cognitive deficits among methamphetamine users with and without past-month psychotic symptoms and a matched sample of healthy controls, we found that MAP was associated with poor emotion recognition, particularly for anger and sadness. In contrast, impairments in emotion recognition were absent in the MNP group, suggesting that deficits in social cognition may be specific to MAP rather than being associated with methamphetamine use per se. Indeed, we found that deficits in steeper delay discounting, which are suggestive of impulsive choices, appear to be more general to methamphetamine use. These differences in cognitive performance were not accounted for by differences in patterns of methamphetamine use, or other potential confounds (age, gender, and IQ).

In contrast with other studies that have investigated emotion recognition in MA-using samples, we specifically examined the influence of psychotic symptoms on performance. In our sample, there were no differences in MA use parameters between the MAP and MNP participants (including age of onset, frequency of use or severity of dependence). As such, our results do not support the concept of emotion recognition deficits as a common correlate of both methamphetamine use and psychosis, but rather, as a more specific correlate of psychotic symptoms in methamphetamine-using individuals.

Our findings are consistent with that of the broader literature of non-drug psychosis, including studies of early-psychosis or first episode psychosis samples where deficits in emotion recognition are evident at first presentation (16, 32). The specific finding of impaired recognition of anger has implications for understanding how people with methamphetamine-associated psychosis interact with others. For instance, this could serve as a mechanism underpinning aggressive behavior in methamphetamine-using populations which has been reported in previous studies (33). Positive psychotic symptoms have an established association with violence (34) and if this is associated with poorer emotion recognition in methamphetamine users, this could lead to misinterpretation of threat, resulting in individuals responding pre-emptively in an aggressive manner to benign social stimuli (33). Importantly, there is a dearth of evidence to guide de-escalation for aggression in psychosis, with a recent Cochrane review failing to identify any trials in this area (35). Our finding of poorer recognition of anger in relation to psychotic symptoms in methamphetamine-using individuals has important clinical implications for treatment providers in emergency and acute health settings, where particular attention may need to be paid to non-verbal and facial communication skills to support more effective de-escalation.

These findings suggest that psychotic symptoms may play a role in influencing social cognition in people who use MA and provide preliminary insights into the relationship between social cognition and methamphetamine-associated psychosis. Although the cross-sectional design was appropriate for between-group comparisons, other limitations of this study design are relevant (36), and we were unable to confirm the direction of association between cognitive impairment and psychotic symptoms. It is possible that impairments in cognition (including deficits in emotion recognition) pre-existed methamphetamine use and/or psychosis, reflecting a vulnerability to psychosis in this population. In this case, the presence of facial emotion recognition deficits may serve as a marker for psychosis vulnerability amongst people who use methamphetamine, and hence may be useful in identifying peopl e who would benefit from early intervention for psychosis. Conversely, it is possible that these social cognition deficits are a consequence of MAP, for example, as neuroadaptation associated with MA use may lead to cognitive impairment and psychosis (17). It is also thought that the process of psychosis itself may lead to cognitive impairment, and this would indicate that the prevention of MAP (e.g., through harm reduction and drug treatment) may also attenuate the cognitive deficits associated with chronic methamphetamine use. Further research to elucidate the chronology of cognitive deficits in relation to MAP may help reveal whether impaired social cognition is a vulnerability marker or a consequence of psychosis.

In line with recent evidence from a meta-analysis of cognition in methamphetamine dependence (17), we found performance on reward-based decision making was impaired in methamphetamine users (both MNP and MAP groups), whereas verbal memory performance was similar across all three groups. There was a significant difference in impulsivity (delay discounting) between the MNP and control group, whereas only trend-level differences were noted between MAP and control participants, suggesting that the presence of psychotic symptoms may decrease impulsivity. Heightened impulsivity has been characterized across substance use disorder groups, particularly in those with stimulant and opioid use disorders, and this may represent a premorbid trait or a consequence of substance use itself (37). Greater impulsivity has a demonstrated impact on clinical outcomes in methamphetamine-using adults, predicting poorer engagement in treatment in early recovery, and poorer quality of life (38, 39).

Strengths of this study included the use of a diagnostic interview (SCID I/P) to exclude pre-existing psychotic disorders, strengthening the interpretation that the symptoms observed in the sample were related to methamphetamine use. This is a key difference in comparison to a substantial number of studies in this area (40). We utilized the Brief Psychiatric Rating Scale (BPRS) (18), a well validated dimensional psychotic symptom measure that has been widely used in other studies of methamphetamine-associated psychosis (19, 41) and primary psychotic disorders (42).

A limitation of the study was that we did not diagnose methamphetamine-induced psychotic disorder, and we did not distinguish between symptoms that were limited to periods of acute intoxication and those that occurred otherwise, so we cannot assume that psychotic symptoms co-occurred with methamphetamine use. However, given the almost-daily patterns of methamphetamine use reported in our sample, and considering that past research has found a strong temporal relationship between methamphetamine use and symptoms of psychosis (19), it is highly probable that symptoms were concurrent with methamphetamine use in most cases. In addition, the study did not include biological verification of methamphetamine use. This approach is consistent with that used in other studies of similar populations, and self-report has been found to be a valid and reliable indicator of drug use, particularly when there is no perceived gain or benefit associated with under-reporting of drug use (43, 44), and the Timeline Followback method used in our study has demonstrated concordance with urinalysis for amphetamines (23).

Being a naturalistic study, participants engaged in the use of other substances, most often cannabis, alcohol and prescription drug use, which could have impacted on cognitive performance. Although there were no differences between measures of alcohol and cannabis dependence between the MAP and MNP groups, non-dependent patterns of substance use may have contributed to impairments in cognitive performance. This is particularly relevant for alcohol use which is shown to impact on emotion recognition (45). Although participants were requested to abstain on the day of the assessment and were seen by clinically-trained researchers experienced in assessing signs of intoxication, we cannot completely exclude the possibility that unmeasured confounds, including acute intoxication, were responsible for cognitive impairments. Finally, although we assessed general cognitive ability using IQ, we did not have a measure of pre-morbid intelligence which may have provided a better measure of this potential confound.

## Conclusion

Although there is a growing body of evidence that stimulant-using individuals present with impairment in social cognition (36, 46), we have shown that such deficits are related to experiencing psychotic symptoms within the past month. However, whether this is a vulnerability marker or a consequence of psychosis requires further elucidation. Nevertheless, these findings contribute to furthering our understanding of the MAP phenotype, and its overlap with other psychotic disorders, as well as having implications for the clinical management of people with this condition.

## Data Availability Statement

The datasets generated for this study will not be made publicly available. Consent was not provided for public availability of data.

## Ethics Statement

The studies involving human participants were reviewed and approved by Monash University Human Research Ethics Committee (CF15/40- 2015000222). The patients/participants provided their written informed consent to participate in this study.

## Author Contributions

SA, AV-G, RM, and DL contributed to study design and protocol. SA, AR and RF contributed to data collection and analysis. SA drafted and refined the manuscript. AV-G, RM and DL provided supervision of the study and edited the manuscript. All authors have reviewed the final version of the manuscript.

## Funding

Author SA was a recipient of a National Health and Medical Research Council (NHMRC) postgraduate scholarship (GNT1093778).

## Conflict of Interest

The authors declare that the research was conducted in the absence of any commercial or financial relationships that could be construed as a potential conflict of interest.
